# Validation study of the prototype of a disease-specific index measure for health-related quality of life in dementia

**DOI:** 10.1186/1477-7525-10-118

**Published:** 2012-09-25

**Authors:** Carla J M Schölzel-Dorenbos, Alexander M M Arons, Joost J G Wammes, MarcelGMOlde Rikkert, Paul F M Krabbe

**Affiliations:** 1 Multidisciplinary Memory Clinic Slingeland Hospital/Alzheimer Centre Nijmegen, Radboud University Nijmegen Medical Centre, Kruisbergseweg 25, Doetinchem, 7009 BL, The Netherlands; 2 Dept. of Epidemiology, Biostatistics & HTA, Radboud University Medical Centre, P.O. Box 9101, Nijmegen, 6500 HB, The Netherlands; 3 Department of Epidemiology, Biostatistics & HTA, Radboud University Medical Centre, P.O. Box 9101, Nijmegen, 6500 HB, The Netherlands; 4 Alzheimer Center Nijmegen, Dept. of Geriatrics, Radboud University Medical Centre, 925 Geriatrics, P.O. Box 9101, Nijmegen, 6500 HB, The Netherlands; 5 Department of Epidemiology, University of Groningen University Medical Center Groningen, PO Box 30.001, Groningen, 9700 RB, The Netherlands

**Keywords:** Dementia, Health-related quality of life, Dementia Quality of life Instrument (DQI), Disease-specific index instrument, Cost-effectiveness

## Abstract

**Background:**

Index measures for health-related quality of life (HRQoL) quantify the desirability (utility) of a certain health state. The commonly used generic index measure, e.g. EuroQol: EQ-5D, may underestimate relevant areas of specific diseases, resulting in lower validity. Disease-specific index measures on the other hand combine disease-specificity and quantification of perceived quality on several health domains of a certain disease into one single figure. These instruments have been developed for several diseases, but a dementia-specific HRQoL index instrument was not yet available. Facing the increasing individual and societal burden of dementia, specific HRQoL values with metric characteristics are especially useful because they will provide vital information for health outcome research and economic evaluations.

**Aims of the study:**

To develop and validate the prototype of a dementia-specific HRQoL index measure: Dementia Quality of life Instrument (DQI), as the first step towards valuation of the dementia health state.

**Methods:**

For development of the DQI we created a conceptual framework based on a review of the literature, qualitative interviews with people with dementia and their carers, expert opinion and team discussion. To assess validity we undertook a survey under 241 dementia professionals. Measurements consisted of ranking (1–5) and rating (1–10) of 5 dementia-specific DQI domains (memory, orientation, independence, social activities and mood) and simultaneously rating of 9 DQI-derived health states on a visual analogue scale (VAS). We also performed a cross-sectional study in a large sample of people with very mild to moderate dementia and their caregivers (N = 145) to assess feasibility and concurrent validity. In addition, caregivers valued 10 DQI and 10 EQ-5D + C derived health states of the patient simultaneously on the same VAS. Setting: outpatient clinics, nursing homes and patient residences.

**Results:**

All professionals judged the selected DQI domains to be relevant. Differences in ranking and rating behaviors were small. Mood was ranked (≥3.3) and rated (≥8.2) as most, orientation as least important (rank ≤2.6, value 7.5) health domain for dementia. For the validation part of this study the completion rates for all domains were above 98% for patients and 100% for caregivers on patients. A priori hypothesized DQI versus QOL-AD correlations that were significant in both patients and caregivers were: memory/memory, orientation/memory, independence/physical health, social activities/energy and mood/mood. Patient/caregiver inter-rater agreement was low (K < 0.2) for memory/independence, fair (K 0.2-0.4) for orientation/mood, and moderate (K 0.4-0.6) for social activities. Concurrent validity of the DQI with the EQ-5D + C was moderate. The fact that most of the correlations between the domains of these two instruments were low (≤0.40) showed that both instruments measure different elements of health status. As expected, modest correlations (≥0.40) were observed between corresponding domains of the two instruments.

**Conclusions:**

Professionals judged all domains as relevant. The DQI prototype proved valid and feasible for patients and caregivers and is appropriate for very mild to moderate dementia. The differences in concurrent correlations with generic health status instruments imply that the dementia-specific DQI health domains indeed provide different information. The finding that patient HRQoL measured with the DQI was lower supports this notion. The new DQI shows comparable psychometric properties to the best available dementia-specific (QOL-AD) and generic (EQ-5D + C) measures. Further research is needed to generate values in the general population for each of the possible DQI states and to derive an algorithm that converts the 5 separate DQI domain scores into one single DQI Index score. Introducing the DQI Index will advance dementia-related HRQoL measurement by overcoming the shortcomings of generic and non-index instruments. This will allow more unequivocal interpretation of subjective dementia HRQoL states in dementia research.

## Background

Dementia is a devastating condition for patients and caregivers and a major public health concern due to its increasing incidence. Assessment of meaningful treatment benefits is complex. Many researchers state that cognitive response no longer suffices in anti-dementia trials [1]. There is emerging consensus on the value of patient-reported outcomes such as health-related quality of life (HRQoL) [2]. There are two fundamentally different approaches to measuring HRQoL. The first is the standard ‘questionnaire’ approach, using descriptive or profile instruments [3]. The second is the ‘index’ approach, using preference-based instruments [4,5].

Descriptive instruments summarize multiple domains of health status and are based on classical test theory [6]. A small set of related items covers the content of various health domains and a score for each dimension is generated. One such frequently used generic descriptive instrument is the SF-36 [7]. Examples of descriptive instruments that are used in dementia include the Quality of Life in Alzheimer’s Disease (QOL-AD) and the Dementia Quality of Life Instrument (D-QOL) [8,9].

Index measures quantify multiple health domains into one single metric figure. In the case of HRQoL, index measures quantify the desirability of a certain health state [10]. The generated values, variously called utilities, preferences or weights, are often unambiguous; e.g., a value of 1.0 stands for ‘perfect health’, 0.0 for ‘death’. HRQoL values with metric characteristics are especially useful because they are applicable in health outcome research and economic evaluations. Descriptive tools lack this feature. The EuroQol-5 D (EQ-5D) is the most widely used generic HRQoL index instrument [11,12]. It includes the five dimensions mobility, self-care, usual activities, pain/discomfort and anxiety/depression.

Both descriptive and index instruments have generic and disease-specific versions, based on the extent to which illnesses are covered. Disease-specific instruments target individual diseases or specific health problems, while generic instruments are more universal and cover general health aspects.

Recently, Riepe et al. concluded that current HRQoL-index instruments, which have been useful in other contexts, are ill-suited and insufficiently validated to play a major role in dementia research, decision making and resource allocation [13]. They reported that six cost-effectiveness studies, using quality-adjusted life years (QALY) measurements, were unsatisfactory, and that large gaps existed between published measurements of HRQoL and the quality standards required by guidelines. Their conclusion was supported by the consensus statement of the International Psychogeriatric Association that generic HRQoL index measures, such as the EQ-5D, are not satisfactorily validated in dementia and that this called into question previous health economic analyses [14]. The solution seems to be a disease-specific HRQoL index instrument. Such instruments have been developed for various diseases but not for dementia [15-19]. We therefore designed a dementia-specific index instrument, the Dementia Quality of life Instrument (DQI).

The DQI is a classification system based on the conceptual framework of the EQ-5D. We replaced the generic EQ-5D domains by domains that are better able to describe the health status in dementia. Our paper presents evidence for the construct validity of the DQI by a detailed listing of the steps taken to prove that the chosen domains indeed represent the construct [20]. Additionally, we undertook a survey under dementia professionals on the contents of the instrument. Next, relations to other variables were examined in dementia patients and their informal caregivers by correlating DQI scores with scores from two well-validated quality of life instruments, one generic and one dementia-specific. Finally, we report on the feasibility of the DQI in dementia patients and caregivers.

## Methods

### Development of the DQI

The following specific features and global constraints were formulated beforehand. 1. Classification of the dementia health states should be based on a limited set of key domains to prevent cognitive overload. 2. Each separate domain should consist of a limited number of levels to facilitate rating. 3. All items should be unequivocally understandable. 4. Consistency throughout domains and levels is mandatory. 5. Responses should be uniform as much as possible. The EQ-5D, for which broadly acknowledged valuation procedures are available to elicitate corresponding values, is widely used due to its ease in use: answers to only five questions result in a HRQoL value. The format of the EQ-5D meets the above described criteria and was used as a template. A drawback of the EQ-5D + C is the presence of composite domains, i.e. pain/discomfort and depression/anxiety. Text books on composing questionnaires recommend to avoid composite items [6]. In the design of the DQI this has been deliberately avoided.

For further development of the DQI, a conceptual framework was generated from a review of the literature, qualitative interviews with people with dementia and their carers, expert opinions and team discussions. The first step was to identify the construct and corresponding content. We searched the literature, databases, ProQolid ( http://www.proqolid.com) and systematic reviews on qualifications of HRQoL in dementia, for previously published instruments, and on HRQoL domains considered important in dementia. We also used qualitative and quantitative information from our earlier HRQoL research in Dutch dementia patients and professionals [21]. This generated a pool of potential scale items. The next step was expert evaluation and reduction of items by team discussion. The selected items were subjected to discussion and challenge within the AD-Euro study group to establish an operational consensus on valid items. The AD-Euro study is a multicentre randomized controlled trial (RCT) that aimed to compare (cost-)effectiveness of post-diagnosis treatment and care-coordination of dementia patients-caregiver pairs by memory clinics versus general practitioners [22]. The experts (N = 6; two geriatricians, master of science in nursing, psychologist, psychometrician and epidemiologist) examined the items and selected the best in several rounds [22]. After each round, a summary from the previous round was provided and judged again. Finally, consensus was achieved in a group meeting resulting in a set of domains judged to fulfill content validity criteria.

### Participants

Professionals were eligible for this validation study if they were working regularly with dementia patients in the field of diagnosis, care, treatment, coordination, and/or counseling. Professionals were divided in subgroups, namely clinical geriatricians (and residents), elderly-care physicians, nurses/nursing assistants and social workers/psychologists. Participants were recruited after a brief introduction during a national conference and by mail through the secretary of their professional associations.

Additionally, 145 pairs of community-dwelling dementia patients and their informal caregivers participating in the AD-Euro RCT (ClinicalTrials.gov NCT00554047) were included in the current study, by performing a cross-sectional analysis of data at T = 6 months. The AD-Euro study which studied follow-up directly after diagnosis recruited 175 patient-caregiver dyads, who visited a multi-disciplinary memory clinic (MMC) specialist, and followed them for a 1 year period. Inclusion criteria were: dementia fulfilling DSM-IV-TR criteria [23], Clinical Dementia Rating (CDR; 0–3) scale score of 0.5-2 (0 for none, 0.5 for questionable/very mild, 1 for mild, 2 for moderate and 3 for severe dementia) [24]. Patients were excluded if 1) their life expectancy was less than 1 year, 2) they were living in a nursing home or already evaluated as being suitable for living in a nursing home, 3) data collection was difficult (e.g. due to severe visual, hearing or language impairment, mood disorder or behavioral disturbances), 4) the patient’s general practitioner did not agree to participate, 5) they were already participating in another study or visited the MMC for a second opinion, or 6) they had a definite indication for MMC follow up. In addition to the CDR, the Mini-Mental State Examination (MMSE) was administered, although scores on this instrument were not an inclusion or exclusion criterion. For further details regarding the AD-Euro study we refer to Meeuwsen et al [22]. Data was collected by trained interviewers, who administered the questionnaires (paper format) and the response tasks at the patient’s home. Interviews were planned in advance with both the patient and the proxy so that data collection occurred simultaneously. The measurements were performed by research assistants, who were blinded to group allocation. The tests were conducted according to the appropriate instructions to the instrument.

### Validation of the DQI

#### Validation of DQI domains

The survey among professionals consisted of three tasks. Task 1 was ranking and Task 2 was rating the domains of the DQI. For Task 1, we asked respondents to choose the order of importance of the domains for dementia patients, from 1 (least important domain) to 5 (most important). For Task 2, respondents rated each separate domain. The assigned value varied between 1 and 10. A value of 1 meant that this domain is totally invaluable, 10 that it is very valuable for dementia patients. Although the valuation task is more informative, ranking can provide additional information over valuations, especially when the domains are more or less equally judged or when respondents are not capable to perform the more difficult valuation task. For Task 3 respondents valued nine dementia health-states, each consisting of a DQI domain combined with one out of the three levels of severity of impairment, on a visual analogue scale (VAS) with poles ranging from 0 (worst imaginable health) to 100 (best imaginable health). These hypothetical states were created in such a way that they largely covered the total spectrum of dementia severity. For the patients’ perspective, this task provided insight in the agreement of health state valuation between patients and proxies [10], as well as an indication whether a ranking task might be a feasible method of health-state utility elicitation.

A similar procedure to Task 3 was performed among caregivers. They valued the 9 DQI health states, as well as the patient’s DQI health state, on the VAS. In addition to this, they also valued 9 hypothetical EQ-5D states as well as the patient EQ-5D state (as caregivers indicated it to be) on the VAS.

#### Validation of DQI outcomes

Concurrent validity for the DQI was examined among caregivers by correlating the scores of the DQI with scores of two well-validated quality of life instruments, one generic and one dementia-specific. The generic instrument was the EQ-5D + C [25-27], and the dementia-specific measure the Quality of Life-Alzheimer’s Disease (QOL-AD) scale [9]. The EQ-5D + C is an extended version of the EQ-5D descriptive system with an additional cognitive domain.

### Statistical analysis

For data analyses of the professionals, Kruskal-Wallis tests were performed to examine differences in ranking behaviors. Different rating behaviors for the separate health domains were assessed with one-way ANOVAs. The same analysis was used to explore possible differences in rating behaviors for the assessment of the constructed DQI health states. Additional Tukey post-hoc tests were performed to examine professional sub-group differences.

To examine the concurrent validity, Spearman rank correlations were calculated between DQI and EQ-5D + C [26,27], and between DQI and QOL-AD [9]. It was hypothesized that the following DQI (higher score = worse HRQoL) versus EQ-5D + C (higher score = worse HRQoL) scores on similar domains would show positive (correlation coefficient *ρ >* 0.2) and significant (*P* < 0.05) correlations: memory/cognition, orientation/cognition, independence/self-care, independence/usual activities, independence/cognition, social activities/usual activities, mood/pain-discomfort, and mood/anxiety-depression. Furthermore, it was hypothesized that the following DQI (higher score = worse HRQoL) versus QOL-AD (higher score = better HRQoL) correlations were negative correlations (*ρ >* 0.2) and that they would be significant (*P* < 0.05): memory/memory, orientation/memory, independence/physical health, independence/ability to do chores around the house, social activities/energy, social activities/ability to do things for fun, and mood/mood. Additionally, it was hypothesized that correlations on patient-data would be lower than caregiver-data, because of the cognitive effects of dementia.

VAS scores of the patients’ health state on the DQI and EQ-5D as assessed by the caregiver were compared by means of a paired sample *t*-test. A significant difference between the average scores of the patients’ health states would be interpreted as discriminative validity of the DQI.

Data were analyzed using SPSS (version 17; SPSS, Inc., Chicago, IL). Patient-caregiver inter-rater agreement was examined by quadratic-weighted Kappa coefficients. Feasibility of the DQI, EQ-5D + C and the QOL-AD were assessed by a missing values analysis. Descriptive statistics were used to examine characteristics at baseline.

## Results

### Design of the DQI

The first step in the design of the DQI consisted of selection of the most relevant domains for dementia, as described above. By consensus five domains (Table 1) were finally selected, which were deemed to comply with the formulated constraints and criteria: memory; orientation (in time and/or place); independence (in daily activities); (engagement in) social activities; and mood.

**Table 1:** Selection of domains for Dementia Quality of life Instrument (DQI)

**Source**	**Memory**	**Orientation**	**ADL**	**IADL**	**Independ.**	**Judgment**	**Probl. solving**	**Social funct.**	**Relat.-ships**	**Self–esteem**	**Mood**	**Affect**	**Anxiety**	**Behavior**	**Well-being**	**Health**	**Hobby**
GINO/CIZ [30]		+	+		+			+	+					+	+		
CDR [24]	+	+	+	+		+	+		+								+
NPI [31]											+		+	+			
Patients [21,32,33]					+				+	+	+	+				+	
Professionals [21,32,33]					+				+	+		+					
Literature [21,32,33]									+	+	+	+				+	
GDS [34]	+	+	+	+									+	+			
CRBRS [35]	+	+	+	+									+	+			
GAS [36]	+		+	+				+						+			+
PGC [37]											+		+				
DEMQOL [38]	+	+	+	+					+	+					+	+	
EQ-5/6D [11,27,39]	+		+	+							+		+				
CIBIS [40]	+	+	+	+		+	+	+			+			+			
IDDD [41]	+	+	+	+	+		+	+									

Abbreviations: *ADL*: Activities in Daily Living. *IADL*: Instrumental *ADL*. Independ.: independency. Probl.: problem. Funct.: functioning. Relat. ships: relationships. *GINO/CIZ*: Geïntegreerd Informatienetwerk Ouderenzorg/Centrum Indicatiestelling Zorg. *CDR*: Clinical Dementia Rating scale. *NPI*: NeuroPsychiatric Inventory. *GDS*: Global Deterioration Scale. *CRBRS*: Crichton Royal Behavioural Rating Scale. *GAS*: Goal Attainment Scaling. *PGC*: Philadelphia Geriatric Center (PGC) Morale Scale. *DEMQOL*: DEMentia Quality Of Life. *EQ-5/6D*: EuroQol-5/6 dimensions. *CIBIS*: Clinician Interview Based Impression of Severity. *IDDD*: Interview of Deterioration of Daily living in Dementia.

**Figure 1:**
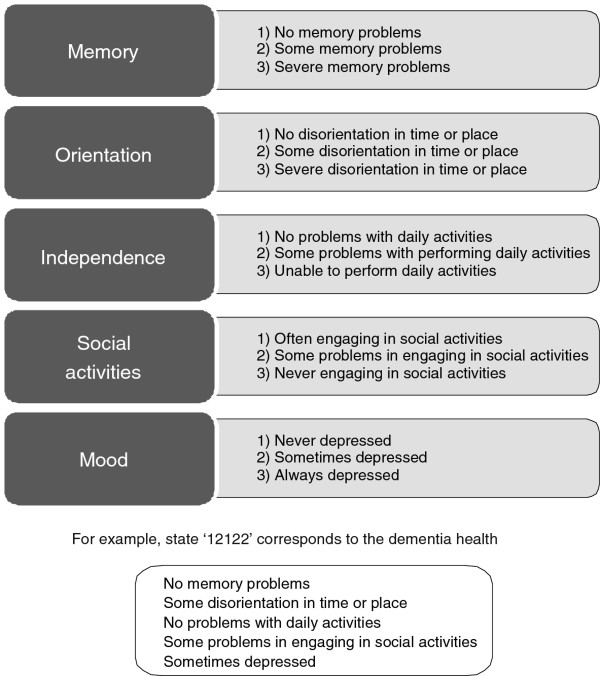
DQI health states (prototype): combinations of five health domains and three levels of severity.

Next, the present status of the patient on these five domains was formulated as a simple statement. The resulting descriptions were combined with one of three severity levels: level 1 = no problems; level 2 = some problems; level 3 = extreme problems. Thus, 11111 represents the best dementia health state, 33333 the worst. Theoretically, this set of five domains and three levels allows for 243 (3 [5]) different health state descriptions across domains and stages relevant in dementia [11]. This resulted in the prototype of the DQI (Figure 1). The second part of the DQI consisted of a visual analogue scale (VAS). This is a vertical 200 mm ‘thermometer’, with 0 indicating the worst imaginable health state and 100 indicating the best imaginable health state. 

### Baseline characteristics

The mean age of the 241 professionals varied between 37 ± 8 years (clinical geriatricians) and 48 ± 8 years (elderly-care physicians). Nurses were 42 ± 2 years of age, nursing assistants 39 ± 10, and social workers/psychologists had a mean age of 44 ± 13 years. Almost two-thirds were nursing assistants (N = 77) or nurses (N = 70). Almost one-third of the participants were physicians: 21% clinical geriatricians and 11% elderly-care physicians. A smaller fraction consisted of social workers/psychologists (together 7% of total). All participants were working in general hospitals or nursing homes. The majority of professionals were female (88% of total, 77-96% of the various subgroups).

The mean age of the patients varied between 80 ± 6 years, 58% were female. Alzheimer’s disease was the most prevalent diagnosis (62%), followed by mixed dementia (28%), vascular dementia (6%) and other (4%). Average patient CDR-scores were 1.1 (SD 0.41), consistent with mild dementia and mean patient MMSE scores were 22.1 (SD 4.3). Patient-caregiver relationships were defined as partners (57%), children (37%) or other (6%). Caregivers were 66 ± 13 years of age, 71% were female.

### Validation of DQI domains in professionals

#### Task 1: Domain ranking task

Ranking of the domains showed for the total group that mood was ranked as the most important health domain for dementia patients, followed by independence. Social activities, memory, and orientation were judged as less important. However, absolute differences were rather small (Table 2).

**Table 2:** Results of ranking and rating of Dementia Quality of life Instrument (DQI) domains by professionals

**Ranking task; Range: 1 (lowest importance) to 5 (highest importance)**					
	Memory	Orientation	Independence	Social activities	Mood
Total group	2.55*	2.30	3.68	2.69	3.78
Nurses	2.77	2.39	3.86	2.66	3.31
Nursing assistants	2.66	2.39	3.14	2.54	4.26
Geriatricians and residents	2.52	2.00	4.02	2.88	3.61
Elderly-care physicians	1.69	2.19	3.96	3.04	4.11
SW^†^ and psychologists	2.44	2.61	3.89	2.44	3.56
**Rating task; Range: 1 (not valuable) to 10 (very valuable)**					
	Memory	Orientation	Independence	Social activities	Mood
Total group	7.2 (2.0)^‡^	7.0 (1.6)	8.2 (1.5)	7.7 (1.3)	8.5 (1.4)
Nurses	7.9 (1.5)	7.5 (1.4)	8.5 (1.4)	7.8 (1.3)	8.3 (1.4)
Nursing assistants	6.9 (2.3)	6.9 (1.5)	7.5 (1.5)	7.6 (1.4)	8.9 (1.1)
Geriatricians and residents	7.0 (1.9)	6.6 (1.8)	8.4 (1.4)	7.6 (1.1)	8.2 (1.4)
Elderly-care physicians	6.7 (1.8)	6.9 (1.4)	8.4 (1.6)	7.7 (1.5)	8.8 (1.0)
SW and psychologists	7.4 (1.7)	7.5 (1.5)	8.6 (1.6)	7.7 (1.0)	8.6 (1.2)

* Mean.

† Social workers.

‡ Mean (Standard Deviation).

We found differences in ranking behavior between subgroups of professionals. The mean ranking values varied from 4.26 (more important) for mood by nursing assistants to 1.69 (less important) for memory by elderly-care physicians. Significant differences (*P* < 0.05) were found for memory, independence, and social activities. Elderly-care physicians ranked memory as the least important domain while nurses ranked it as most important. Independence was ranked least important by nursing assistants.

#### Task 2: Domain rating task

The results of the domain rating task of the total group of professionals showed exactly the same ordering as found for the ranking task (see Table 2). Scores were highest (most valuable) for mood (8.5) and lowest for orientation (7.0). Rating behaviors differed between subgroups for memory, orientation and independence. Memory and orientation were judged more valuable by nurses, and nursing assistants judged independence less valuable. Differences in rating behavior on the other domains were non-significant. Results of comparisons between subgroups on rating as the least or most valuable domain showed significant differences (*P* < .05) for memory, orientation and independence.

#### Task 3: Health state valuation task

**Figure 2:**
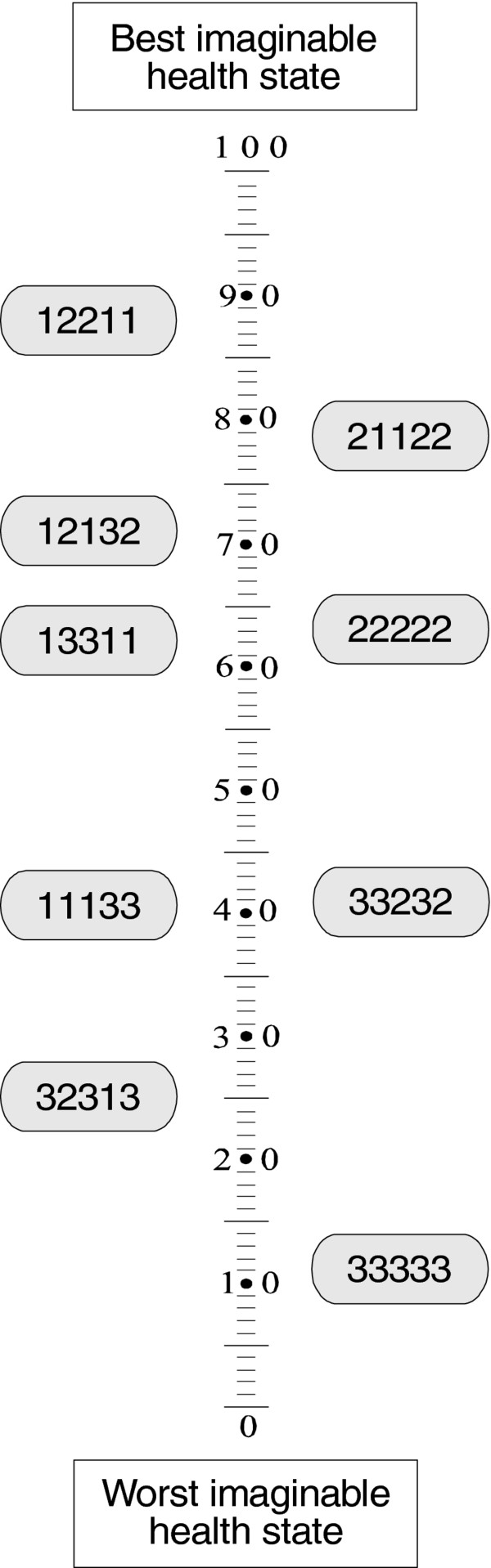
Valuation (Task 3): valuing of 9 DQI health states on a visual analogue scale by professionals.

This task showed that DQI state 33333 was valued lowest with a value of 11.3 on the VAS (0–100) whereas DQI state 12211 was valued as the best state with a value of 88.4 (Figure 2).

Significant differences in values between the subgroups of professionals were observed for states 12211, 21122, 12132, 22222, and 11133 (all *P* < 0.05). For all these hypothetical health states, both nursing assistants, nurses valued these dementia states as better compared to other subgroups of professionals. There was a significant difference between the average DQI patient health state and the average EQ-5D patient health state, as indicated by caregivers.

### Validation of DQI outcomes in patients and caregivers

The a priori hypothesized DQI versus EQ-5D + C correlations that were significant in both patients and caregivers were: memory/cognition, orientation/cognition, independence/self-care, independence/usual activities, independence/cognition and mood/depression-anxiety (Table 3). Correlations that were hypothesized a priori but were not significant for patients were: social activities/usual activities and mood/pain-discomfort. These correlations were significant for caregivers.

**Table 3:** Concurrent validity (correlations) between Dementia Quality of life Instrument (DQI) and EQ-5D + C in patients and caregivers

**EQ-5D + C domains**	**DQI domains**					
	**Memory**	**Orientation**	**Independence**	**Social activities**	**Mood**	**N**
Assessment of caregivers on patients						
Mobility	0.27**	0.13	0.23**	0.07	0.11	145
Self-care	0.26**	0.19*	0.42**	0.05	0.18*	145
Usual activities	0.36**	0.25**	0.46**	0.22**	0.15	145
Pain/discomfort	0.12	0.17*	0.06	−0.09	0.18*	145
Depression/anxiety	−0.04	0.01	0.05	0.12	0.50**	145
Cognition	0.49**	0.36**	0.39**	0.11	0.19*	143
Self assessment of patients						
Mobility	0.24**	0.09	0.22**	0.09	−0.06	139
Self-care	0.22**	0.20*	0.30**	0.10	0.01	139
Usual activities	0.17*	0.20*	0.29**	−0.02	0.15	140
Pain/discomfort	0.10	0.19*	−0.03	−0.01	0.01	140
Depression/anxiety	−0.05	0.01	0.03	−0.13	0.20*	140
Cognition	0.35**	0.32**	0.20*	−0.09	0.05	139

*Correlation is significant at the 0.05 level (2-tailed).

**Correlation is significant at the 0.01 level (2-tailed).

A priori hypothesized DQI versus QOL-AD correlations that were significant in both patients and caregivers were: memory/memory, orientation/memory, independence/physical health, social activities/energy and mood/mood. Correlations that were hypothesized a priori but not statistical significant for patients were: independence/ability to do chores around the house and social activities/ability to do things for fun. These correlations were statistically significant for caregivers. Patient/caregiver inter-rater agreement was low (K < 0.2) for memory and independence, fair (K 0.2-0.4) for orientation and mood, and moderate (K 0.4-0.6) for social activities.

Feasibility of the DQI was assessed by completion rates. All five domains had a completion rate of above 98.6% for patients, whereas for caregivers the completion rate was 100% in all domains. Patient completion rates for the EQ-5D + C were 97.9% for self-care and cognition, 98.6% for mobility and daily activities and 99.3% for pain/discomfort and anxiety/depression. Caregivers had a completion rate of 100% in all domains. Patient completion rates for the QOL-AD were 77.1% for marriage (the low completion rate of this item may be explained by the fact that the question about marriage was often answered with ‘not applicable’), 98.6% for friends and ability to do chores around the house, 99.3% for self as a whole and 100% for the remaining domains. Caregivers had a completion rate of 90.9% for marriage, 99.3% for mood and ability to do things for fun and 100% for the remaining domains.

**Figure 3:**
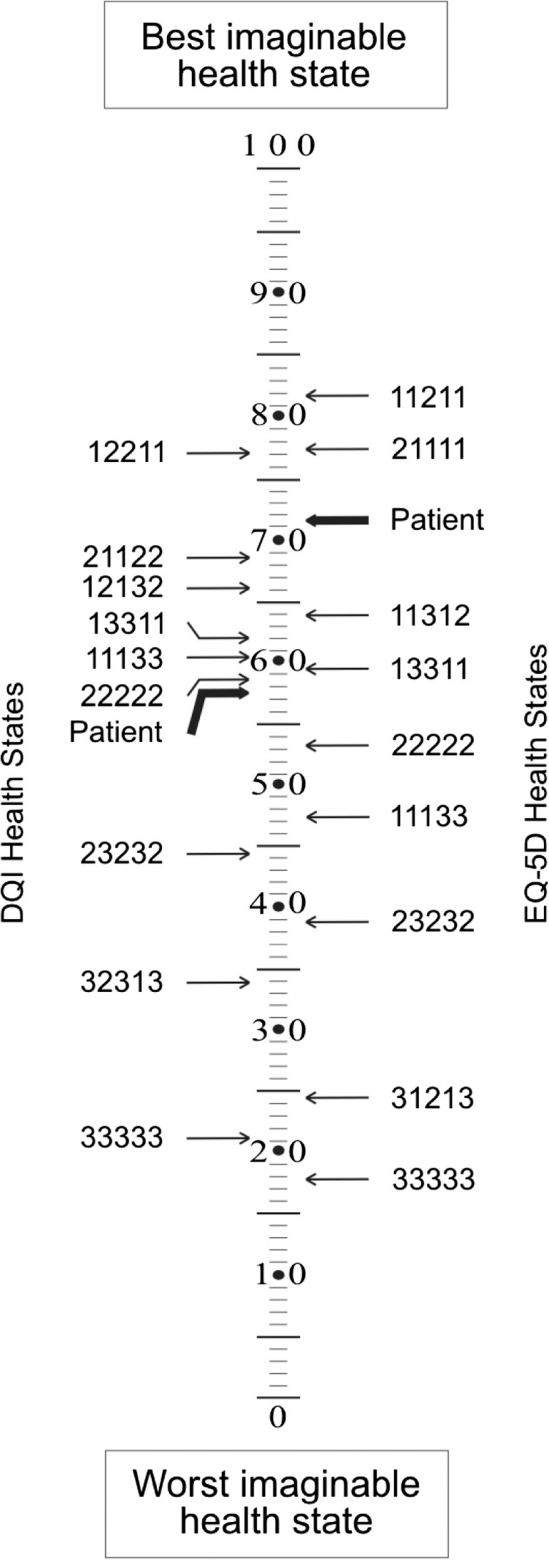
Simultaneous assessment by caregivers of patients’ HRQoL with Dementia Quality of life Instrument (DQI) and EQ-5D*.

The patient health state as classified by caregivers on the DQI was rated statistically significantly lower than the patient health state classified by caregivers on the EQ-5D (difference $\bar{x}=13.69$, SD 21.03, *p* < 0.001, Figure 3).

## Discussion

The present study provides evidence for validity and feasibility of the DQI in dementia, which was based on a literature search, patient information, expert opinion and team discussion, and for format adapted from the widely used generic index instrument EQ-5D. In the subsequent empirical validation and reliability testings, both in professionals and patients, and carers we found that the dementia specific DQI had added value compared to the generic health status quantification with the EuroQol or the descriptive quality of life rating with the QOL-AD.

Our survey under dementia professionals showed that the selected DQI health domains were considered as relevant and important for HRQoL of dementia patients. Overall values were well in the upper range from 1 (not valuable) to 10 (very valuable). Mood was judged as most important and orientation as least important domain. Small differences between professional subgroups could probably be explained by their different professional backgrounds, different types of professional contact, and stage of dementia that they face while working with their patients. In more advanced stages of dementia other needs, priorities and symptoms emerge.

Our concurrent validation study, in dementia patients and in caregivers on patients, showed that the DQI (a dementia-specific HRQoL index instrument) correlated moderately with the EQ-5D + C (a generic HRQoL index instrument) and the QOL-AD (a dementia-specific HRQoL instrument). The fact that most of the correlations between the domains of the EQ-5D + C and the DQI are rather modest shows first of all that each of the separate domains of both instruments are relatively independent. This indicates that the content of the domains reflect different elements of health status. The same holds between the five domains of the two instruments itself. Correlations were highest when the domains were (nearly) identical between the instruments. The differences in correlations imply that the dementia-specific DQI health domains indeed provide different information than the generic EQ-5D + C health domains. The finding that patient HRQoL measured with the DQI was lower than measured by the EQ-5D + C supports this notion. No statistically significant correlation was observed between the mood domain of the DQI and the pain/discomfort domain of the EQ-5D + C. This may be a result because these are different constructs’.

In our validation studies, caregiver correlations were higher than patient correlations. This can probably be attributed to the cognitive effects of dementia. Nevertheless, patient-caregiver inter-rater agreement was fair on average and the results are in line with other instruments used with dementia patients and caregivers [28,29].

The feasibility of the DQI was very high and comparable to that of the EQ-5D + C and the QOL-AD. Nearly all patients and all caregivers were able to complete the instrument. Therefore, we conclude that the DQI performs well for evaluating HRQoL in a mild to moderate dementia population.

Our next step is to convert the DQI prototype into the final version of the DQI and to generate values for each of the possible DQI health states. These values will be generated in the general population, with sufficient older persons, to derive an algorithm that converts the separate DQI domain scores into one single DQI index score. This metric figure will enable unequivocal interpretation of subjective dementia HRQoL states. The DQI Index is the ‘raison d’être’ for the DQI. The EQ-5D does provide HRQoL values, but is too generic (lacks content validity) to acknowledge the specific problems of dementia. The QOL-AD and other similar instruments (e.g. D-QOL) are dementia-specific, but have only been developed to produce a sum score for a set of separate domains. The DQI Index will advance HRQoL measurement in dementia by overcoming both these shortcomings, and therefore provide the field with an outcome measure of added value for evaluation research in dementia.

## Competing interests

All authors declare that they have no competing interests.

## Authors’ contributions

All authors contributed to all aspects of this paper. The corresponding author had full access to all the data in the study and had final responsibility for the decision to submit for publication. Concept and design of DQI: CSD, PK. Design of survey: CSD, MOR, PK. Analysis data: AA, PK, JW. All authors read and approved that final manuscript.
